# Adapting Pediatric Emergency Services for Children with Autism Spectrum Disorder: A Phenomenological Approach

**DOI:** 10.3390/children12091275

**Published:** 2025-09-22

**Authors:** Saray Betancort-Avero, María-Ángeles Ferrera-Fernández, Héctor González-de la Torre, Javier Auyanet-Franchy, Claudio-Alberto Rodríguez-Suárez

**Affiliations:** 1 Complejo Hospitalario Universitario Insular Materno Infantil (CHUIMI), Canary Health Service, 35016 Las Palmas, Spain; sbetave@gobiernodecanarias.org (S.B.-A.); maferferl@gobiernodecanarias.org (M.-Á.F.-F.); jauyfra@gobiernodecanarias.org (J.A.-F.); claudioalberto.rodriguez@ulpgc.es (C.-A.R.-S.); 2 Nursing Department, Faculty of Health Science, University of Las Palmas de Gran Canaria (ULPGC), 35016 Las Palmas, Spain

**Keywords:** autism spectrum disorder, autistic disorder, emergency service, hospital, pediatric emergency medicine, patients’ rooms, health facility environment, qualitative research

## Abstract

**Highlights:**

**What are the main findings?**

- Both healthcare professionals and parents recognize the need for adaptations in pediatric emergency departments to better serve children with Autism Spectrum Disorder.
- Among the proposed adaptations, a sensory-adapted room was highlighted as a valuable resource, while broader systemic changes were also emphasized.
- The main barriers identified include a lack of professional training, inadequate hospital environments, and stress affecting both patients and providers.

**What is the implication of the main finding?**

- Implementing sensory-adapted spaces, together with targeted training programs, can promote safer, more humane, and inclusive care for children with Autism Spectrum Disorder.
- More broadly, adapting pediatric emergency services to neurodiverse needs may improve the experiences of both patients and families while reducing emotional distress during hospital visits.

**Abstract:**

**Background/Objectives**: Children with Autism Spectrum Disorder (ASD) who attend pediatric emergency services face challenges related to their sensory, cognitive, and behavioral characteristics. This study explored the perceptions of healthcare professionals and parents regarding the need to implement adaptations, particularly a sensory-adapted room, for children with ASD in pediatric emergency departments. **Methods**: A phenomenological qualitative study was conducted through semi-structured interviews (October–December 2024) until data saturation. Participants included healthcare professionals and parents of children diagnosed with ASD. Intentional coding and co-occurrence analysis were performed using Atlas.ti (version 25.0.1). The study was approved by the Research Ethics Committee (code: 204-458-1). **Results**: Eighteen informants participated (10 professionals and 8 parents). Professionals’ interviews revealed three themes and eight subthemes: Professional Training (approach strategies; training received; perceived needs), Hospital Environment (resource allocation; infrastructure; perceived needs during the emergency visit), and Emotional Aspects (emotional experience related to patient care; professionals’ personal perceptions). Parents’ interviews yielded four themes and ten subthemes: Professional Training (perceptions of staff training; demonstrated emotional competencies; socioemotional relationships during care), Hospital Environment (infrastructure; perceived needs during emergency visits), Emotional Aspects (families’ experiences; emotions during care), and ASD (diagnostic characteristics; children’s needs; sensory regulation). **Conclusions**: Pediatric emergency services should be adapted to better meet the needs of children with ASD. Both healthcare professionals and parents recognize the importance of such adaptations, particularly sensory-adapted spaces. The main barriers identified were a lack of professional training, inadequate hospital environments, and stress affecting both patients and provider. Priority measures include continuous ASD-specific training programs, improvements in sensory infrastructure, and more flexible clinical protocols, advancing toward a more inclusive and comprehensive model of care.

## 1. Introduction

Autism Spectrum Disorder (ASD) is a neurodevelopmental disorder characterized by persistent difficulties in social communication and interaction, along with restricted and repetitive patterns of behavior, interests, or activities. According to the *Diagnostic and Statistical Manual of Mental Disorders* (*DSM-5*), these symptoms are present from early developmental stages, cause clinically significant impairments in daily life, and cannot be better explained by intellectual disability or global developmental delay [1]. The clinical manifestations of ASD range from deficits in socio-emotional reciprocity, atypical use of body language, and difficulties in understanding or maintaining social relationships to stereotyped behaviors, rigid adherence to routines, intensely focused interests, and altered sensory responses—either hypersensitivity or hyposensitivity [1]. The severity of the disorder varies among individuals and is classified into three levels based on the degree of support required: level 1 (requiring support), level 2 (requiring substantial support), and level 3 (requiring very substantial support). This classification considers both the capacity for social interaction and the rigidity of behavior [1].

In hospital settings, it is essential to design environments that accommodate the specific needs of individuals with ASD to optimize their care experience. However, there is still little consensus regarding the precise meaning of the term “autism-friendly.” To implement an “autism-friendly” model in hospitals, several barriers and facilitators have been identified across three key dimensions: people, place, and time. Flexibility in each of these areas is crucial for improving both care and the overall experience of patients with ASD [2]. In pediatric emergency departments, the hospital environment can be particularly challenging for children with ASD due to heightened sensitivity to sensory stimuli, difficulty tolerating unexpected changes, and limitations in communication skills. These factors often result in severe anxiety, disruptive behaviors, or withdrawal, which complicate clinical assessment, delay care, and generate frustration for both families and healthcare professionals [3,4]. Several studies have highlighted that, in their current configuration, emergency rooms do not adequately address the needs of this population. The literature describes numerous sensory-stimulating elements in these environments that act as barriers to appropriate care. The most frequently reported obstacles include physical factors such as high levels of light and noise [5], the lack of specialized training among healthcare staff [6,7], organizational shortcomings such as the absence of adapted protocols [8], and the scarcity of sensory-regulated spaces [7,9]. Furthermore, the need to improve communication processes, strengthen interprofessional collaboration, and provide greater support for families has been consistently emphasized [6,7,9,10,11].

In response, recent studies have demonstrated the positive impact of sensory-friendly environments in hospital contexts. Interventions such as the creation of sensory rooms, the use of adapted visual and auditory tools, and continuous staff training have shown benefits for both quality of care and the experiences of patients and caregivers [12].

At the time this study was conducted, the Pediatric Emergency Department of the Hospital Universitario Materno Infantil de Canarias (HUMIC) did not have a sensory-adapted space for children with ASD. In this context, it was considered essential to understand, from a phenomenological perspective, how parents and professionals perceive the potential implementation of a sensory room within this department. This qualitative approach aims to explore in depth the experiences and emerging needs related to the healthcare of children with ASD in order to inform proposals that promote more humanized, safe, and effective care for this population. Therefore, the objective of this study was to explore the perceptions of healthcare professionals and parents regarding the need to implement adaptations, particularly a sensory-adapted room, for children with ASD in the pediatric emergency department.

## 2. Materials and Methods

### 2.1. Design

A qualitative study with a phenomenological approach was conducted, combining Husserl’s descriptive perspective [13] and Heidegger’s interpretative hermeneutics [14]. Generative artificial intelligence (GenAI) was not used in the conception, preparation, or writing of this paper.

### 2.2. Experience or Role of Researchers

The research team consisted of five professionals (two women and three men). Four of them (SBA, MAFF, JAF, and CARS) were pediatric nursing specialists, and three held PhDs in Nursing and Health Sciences (MAFF, HGdlT, and CARS) with prior experience in qualitative research. The remaining authors had no prior contact with any of the participants.

### 2.3. Participants and Sampling

The pediatric emergency department is part of HUMIC and provides care to the pediatric population of Gran Canaria. It functions as a tertiary-level monographic hospital and serves as a referral center for the province of Las Palmas and the entire autonomous community of the Canary Islands (Spain). The department is staffed by more than 60 professionals, including pediatricians, pediatric nurses, medical residents (MIR), nursing residents (EIR), and auxiliary staff such as nursing care technicians (TCAE).

Regarding children with ASD, available data estimate a prevalence of 0.61% in the Canary Islands, consistent with figures reported nationally and internationally [15]. Several family associations exist in the region, notably “Con tu ayuda todos sumaremos”, which brings together 74 families.

Participants were recruited through convenience sampling with a snowball strategy, ensuring diversity in sociodemographic profiles to enrich the variability of discourses on the phenomenon under study [16]. Following Hennink et al. [17], nine interviews were considered sufficient to achieve code saturation, while 16 to 24 interviews were required to reach meaning saturation. Therefore, a sample of 20 participants (10 professionals and 10 parents) was deemed appropriate. One researcher (SBA) invited 20 professionals directly via personalized invitations, of whom 10 agreed to participate, and 74 families were invited through the association’s internal communication channel, of whom 10 participated. Eligibility criteria included professionals with at least one year of experience in the HUMIC emergency department and parents of children diagnosed with ASD aged 3–15 years. Families of children diagnosed less than one year prior were excluded. Withdrawal criteria included revocation of informed consent.

### 2.4. Data Collection

Data were obtained through in-depth semi-structured interviews conducted in person by a researcher (SBA) between November and December 2024. Participants were recruited based on experiences within the past twelve months. Interviews were scheduled at mutually convenient times to avoid disruption of work activities. Quiet, comfortable, and interruption-free spaces within the hospital were arranged to minimize unnecessary travel. Relevant sociodemographic variables were collected to contextualize the narratives: for professionals, gender, age, professional category, and years of experience; for parents, relationship to the child, age, employment status, number of children, and age of the child with ASD. Field notes were also maintained to complement the interviews. Information on the specific reasons for pediatric emergency department visits was not available. Interviews lasted 30 to 120 min and followed a semi-structured script adapted for each participant group, as presented in Supplementary Table S1.

### 2.5. Data Analysis

Interviews were audio-recorded (PHILIPS^®^ DVT1160 8GB) and transcribed verbatim immediately after each session. Transcription allowed for progressive analysis, identification of relevant quotations, and initial open coding to generate analytical categories [18]. Analysis followed the Glaser and Strauss approach [19], categorizing information into descriptive codes or meaning units (MU) and grouping them into subthemes until theoretical saturation was reached. Selective coding integrated and refined similar categories, identifying those with stronger empirical grounding as central theoretical categories. Axial coding was then performed through analysis of category co-occurrences, identifying relationships between frequently co-occurring categories to explore interconnections, patterns, and emerging themes [20]. Analysis was conducted using Atlas.ti^®^ (version 25.0.1; Scientific Software Development GmbH, Berlin, Germany), with results presented via verbatim quotations and co-occurrence tables to support both descriptive and interpretative analyses.

### 2.6. Rigor and Trustworthiness

Methodological rigor was ensured following Lincoln and Guba’s criteria [21,22]. Credibility was enhanced through detailed data collection and participant verification of transcriptions. Transferability was addressed through comprehensive descriptions of setting, participants, context, and method. Dependability was evaluated via external review by two experts (CARS and HGdlT) not involved in data collection or analysis. Confirmability was ensured through triangulation of transcripts, field notes, and inter-rater reflection on potential researcher biases. Data triangulation included cross-checking transcripts with field diary notes. The study adhered to the Consolidated Criteria for Reporting Qualitative Research (COREQ) guidelines [23] for quality and transparency in qualitative research.

### 2.7. Ethical Considerations

Confidentiality, anonymity, and exclusive use of data for research purposes were maintained. Data collection, processing, and storage complied with current legislation. Ethical principles from the Nuremberg Code, the Declaration of Helsinki, and the Belmont Report were applied, ensuring informed consent, respect for autonomy, and participant protection. The study was approved by the Research Ethics Committee of Hospital Universitario Doctor Negrín, Gran Canaria, Las Palmas (code: 2024-548-1).

## 3. Results

### 3.1. Participant Characteristics

Of the total participants contacted (20 professionals and 74 parents), 10 professionals and 10 parents initially accepted the invitation. However, two parents did not maintain the necessary contact for inclusion in the study; therefore, the final sample consisted of 18 informants (*n* = 10 professionals and *n* = 8 parents). The sociodemographic characteristics of professionals are presented in Table 1 and those of parents in Table 2.

### 3.2. Themes

A total of 150 verbatims were identified (*n* = 54 professionals and *n* = 96 parents), which were coded into 290 MUs (*n* = 110 from professionals and *n* = 180 from parents).

For professionals, eight subthemes were identified, organized into three main themes. The first theme, Professional Training, included Approach Strategies (*n* = 19 MU), Training Received (*n* = 20 MU), and Perceived Needs (*n* = 17 MU). The second theme, Hospital Environment, encompassed Resource Allocation (*n* = 9 MU), Infrastructure (*n* = 10 MU), and Perceptions of the Need for an Adapted Room (*n* = 14 MU). The third theme, Emotional Aspects, included Emotional Experience Related to Patient Care (*n* = 12 MU) and Personal Perceptions of Professionals (*n* = 9 MU).

For parents, four main themes were identified. The first, Professional Training, included Perceptions of Staff Training (*n* = 18 MU), Demonstrated Emotional Competencies (*n* = 11 MU), and Socioemotional Relationships Established During Care (*n* = 15 MU). The second theme, Hospital Environment, encompassed Infrastructure (*n* = 19 MU) and Perceived Needs During the Emergency Visit (*n* = 37 MU). The third theme, Emotional Aspects, included Families’ Experiences (*n* = 13 MU) and Emotions Experienced During Care (*n* = 27 MU). The fourth theme, Autism Spectrum Disorder, incorporated Diagnostic Characteristics (*n* = 21 MU), Children’s Specific Needs (*n* = 18 MU), and Sensory Regulation (*n* = 16 MU). All themes, subthemes, and MUs are presented in Table 3.

#### 3.2.1. Professionals

- Theme 1: Professional Training

Professionals reported various strategies for approaching children with ASD, including the use of pictograms, familiar objects, sedation, and nonverbal communication:

“*We have pictograms and other distraction methods. The collaboration of the family is essential for the relaxation of these patients*”.

(Professional 06)

“*Speak clearly and slowly, always accompanied by a reference figure for them*”.

(Professional 01)

Regarding training, there was a widespread perception of deficiencies in formal preparation:

“*We have trained ourselves more on our own than through the institution. It is all very self-taught*”.

(Professional 07)

“*Knowledge has been acquired through experience. I have never received training; you learn as you go*”.

(Professional 03)

Professionals also expressed perceived needs related to individualized care, time constraints, and lack of resources:

“*We lack time and staff. You cannot give a child with autism the attention he needs*”.

(Professional 05)

“*Adapted rooms, time to treat and talk to the children calmly … That would be ideal: time, resources, and training*”.

(Professional 09)

- Theme 2: Hospital Environment

Professionals highlighted material limitations and the absence of adapted sensory stimuli:

“*We are not prepared; there is no space for them*”.

(Professional 05)

Infrastructure was also a frequent source of concern:

“*The waiting room is a chaos of stimuli. For a child with ASD, it is hell*”.

(Professional 02)

“*A hostile environment such as the emergency department, where quite anxiety-inducing procedures are carried out*”.

(Professional 06)

Most professionals valued the potential of having an adapted room:

“*A quiet room, with less light and noise, would be ideal. That would make the difference*”.

(Professional 08)

- Theme 3: Emotional Aspects

The accounts revealed a significant emotional impact:

“*Many times, I leave feeling frustrated. I feel that we are not doing things right with these children*”.

(Professional 10)

“*They are patients who move you deeply. They demand more from you as a person than as a professional*”.

(Professional 01)

“… *from the outset they reject physical contact … the child feels cornered. Several of us are going to hold him, inject him, suture him, or perform an electrocardiogram, and they do not understand why*”.

(Professional 03)

Regarding personal perceptions, professionals expressed stress and a sense of helplessness due to lack of preparation:

“*You feel like you are improvising, that you don’t have the tools to do it properly*”.

(Professional 09)

#### 3.2.2. Parents

- Theme 1: Professional Training

Parents expressed a general perception of insufficient training among healthcare personnel and highlighted the importance of professionals adopting understanding and empathetic attitudes:

“*When I have encountered a professional who showed knowledge, it was due to personal experiences. One must be prepared for this type of person*”.

(Parent 03)

“*There is a lack of empathy and knowledge. They are not prepared, and it shows. To all professionals: be attentive, read, observe the situation, put yourselves in our place, and be empathetic*”.

(Parent 06)

“*Since they are not trained and are not aware of the characteristics of children with ASD, what they think is that my son is poorly behaved, and with the looks they give me I feel judged. Mothers notice this; it’s been nine years living through these situations*…”.

(Parent 06)

Some parents also noted a lack of sensitivity:

“*There isn’t even the question ‘Do you need something?’ Nothing, no trace of empathy. We would love not to have a disability, but we do*”.

(Parent 07)

Tensions and judgments regarding parenting were also reported:

“*I felt that I was being blamed for not knowing how to calm my son*”.

(Parent 04)

“*There was little tact; I felt judged as a mother*”.

(Parent 08)

- Theme 2: Hospital Environment

Parents described the infrastructure as inadequate:

“*The waiting room is ridiculous and not functional at all. You only have chargers*”.

(Parent 07)

“*We need a quieter space, with toys or something visual to distract him*”.

(Parent 05)

“*There is nothing aimed at children, not even a table with crayons to draw*”.

(Parent 02)

Regarding perceived needs, parents expressed multiple shortcomings:

“*I don’t see them using visual supports either. They don’t explain things to him before doing them; it’s usually me. It depends on the staff on duty at that moment*”.

(Parent 02)

“*I miss being asked—by the father or the mother—how we can better manage with the children. Each child works differently. At least there should be a little communication*”.

(Parent 03)

- Theme 3: Emotional Aspects

Parents’ experiences ranged from dissatisfaction to emotional overload:

“*The waiting room is crowded, and my son cannot tolerate the babies crying; he gets distressed because he does not know how to comfort them. For me, it is traumatic*”.

(Parent 07)

“*I left there with anxiety. It was more traumatic for me than for him*”.

(Parent 02)

The emotions expressed included frustration, distress, helplessness, and hope:

“*Sometimes the doctor understands and asks for my collaboration, but other times they do not and prefer to do it their way no matter how much I explain. They make the child have a bad time by not listening to me and not letting me hold him. At no point do they address the child; out of ten doctors, two will do so. The rest sometimes do not even explain things to me, and that is frustrating, to say the least*”.

(Parent 04)

- Theme 4: Autism Spectrum Disorder

Families described diagnostic characteristics such as sensory sensitivity, intolerance to noise, and communication difficulties:

“*When my son gets nervous, he flaps his hands and covers his ears… The strategies we use at home to help him regulate are physical contact with me, holding him gently, hugging him, and talking to him about things he likes to distract him*”.

(Parent 03)

Regarding specific needs, parents emphasized the importance of individualized and adapted care:

“*They throw at you the phrase ‘the protocol is like this for neurotypical children.’ Adaptation is lacking; there are children who function differently. A flexible protocol is necessary*”.

(Parent 03)

In relation to sensory regulation, strategies such as the use of technology or visual aids were mentioned:

“*I always bring the cellphone with cartoons; it is the only way to calm him down*”.

(Parent 01)

“*With softer lights and less noise, everything would be much more manageable*”.

(Parent 08)

### 3.3. Co-Occurrences

The co-occurrence analysis of subthemes identified in the professionals’ interviews revealed several significant connections. The most prominent were those linking Approach Strategies with Emotional Experience Related to Patient Care (*n* = 7) and with Perceived Needs (*n* = 7). Notable associations were also observed between Training Received and Perceived Needs (*n* = 5), as well as between Resource Allocation and Perceived Needs During the Emergency Visit (*n* = 5), as presented in Table 4.

Regarding parents, the most prominent co-occurrences were concentrated around the subtheme Emotions During Care, particularly in relation to Families’ Experiences (*n* = 22), Diagnostic Characteristics (*n* = 13), Perceived Needs During the Emergency Visit (*n* = 17), and Infrastructure (*n* = 13). Notably, the subtheme Perceived Needs During the Emergency Visit showed high co-occurrence with most other subthemes, being especially significant in its associations with Diagnostic Characteristics (*n* = 13), Families’ Experiences (*n* = 12), Perceptions of Staff Training (*n* = 11), and Infrastructure (*n* = 11), as presented in Table 5.

## 4. Discussion

The findings highlight an urgent need for transformation in pediatric emergency services to improve the quality of care for children with ASD. Consistent with previous literature, the chaotic nature of the hospital environment, combined with the lack of sensory and communication adaptations, was observed to create a distressing experience for both patients and their parents [6,24]. In particular, waiting rooms were identified as critical spaces where sensory overstimulation and prolonged waiting times can trigger disruptive behaviors and increase the likelihood of more invasive clinical interventions [7].

With regard to professional training, both healthcare professionals and parents agreed that preparation for caring for children with ASD is insufficient and poorly structured. Professionals reported that, although they often use strategies such as pictograms, familiar objects, or nonverbal communication, much of their knowledge has been self-taught and gained through experience, highlighting the marked absence of systematic institutional training programs [25,26]. This training gap has been widely acknowledged in the literature, which emphasizes that the lack of formal ASD training in hospital settings contributes to insecurity, frustration, and stress among healthcare staff [27,28]. Parents, in turn, perceive this lack of knowledge and empathy, which negatively affects trust and the quality of the relationship with professionals [24]. These findings are consistent with those reported by Nicholas et al. [6], who underscore the urgent need for continuous training programs focused on sensory care, behavioral management, and adapted communication strategies.

In line with the observations of Ozturk and Merter [29], the physical infrastructure of the hospital environment is identified as one of the main obstacles to providing safe care tailored to the needs of children with ASD. The absence of sensory-adapted spaces, together with the rigidity of care protocols, may challenge the ability of healthcare staff to deliver individualized and humanized care [29,30]. This is further compounded by the scarcity of adaptive materials, such as sensory toys, pictograms, and communication support tools, which widens the care gap. These findings are consistent with those of Gormley et al. [30], who highlight the importance of implementing augmentative communication systems to improve the care of patients with language difficulties. From the families’ perspective, noisy, chaotic, and unwelcoming hospital environments—particularly waiting rooms—increase anxiety and can generate feelings of loneliness, disorientation, and abandonment [24]. The specialized literature emphasizes that investment in sensory-adapted infrastructures not only reduces anxiety and improves the hospital experience but also promotes patient cooperation and efficiency of care [6,31,32].

The emotional dimension emerged as a central axis in the experiences of both professionals and families. Professionals reported feelings of frustration, stress, and helplessness, particularly related to the lack of material resources and specific training. Families, in turn, expressed intense emotions such as anxiety, distress, and a sense of abandonment, although they also noted moments of hope and relief when care was empathetic, respectful, and adapted to their needs [24]. These findings are consistent with Ben Natan et al. [33], who highlight the profound emotional impact that the healthcare system can generate and the need to implement emotional support strategies to improve the care experience.

Research indicates that the communicative difficulties of children with ASD directly influence parents’ perception of care quality, particularly regarding waiting times, staff responsiveness, coordination among professionals, and the overall organization of services. The study also confirms that both mothers and fathers experience complex emotional processes and face similar practical challenges, including stages of grief, the need for clear information, sustained emotional support, sufficient economic resources, and effective family teamwork. These similarities reinforce the importance of designing clinical interventions that actively consider both parents as recipients of support and guidance.

Nurses play a key role within the healthcare system, acting as a bridge between families and the healthcare team. They provide active listening, emotional support, practical guidance, and education about ASD. Tools such as informational booklets or accessible digital resources facilitate access to information, improve family coping, and reduce caregiver burden [34]. However, a concerning disconnection between some professionals and caregivers was also identified, with parents sometimes feeling unheard or blamed for their children’s behaviors during emergency visits [6,24]. This communication gap undermines mutual trust and hinders effective collaboration, which is essential to improving the quality and safety of care [29]. The specific characteristics of ASD demand a highly personalized and flexible approach, where sensory regulation, communicative adaptation, and respect for the child’s pace are fundamental [24]. Both families and professionals emphasized the importance of implementing adapted strategies such as visual aids, minimization of environmental stimuli, and predictable routines—key elements to avoid sensory overload and enhance the child’s cooperation during care [6,24]. The literature supports these needs, highlighting the effectiveness of “sensory-friendly” care models and non-pharmacological interventions to foster positive, humanized, and safe experiences for children with ASD [7,32]. Care model inspired by Jean Watson’s Theory of Human Caring, as adapted by Wood et al. [7], demonstrate that transforming the physical environment, combined with interdisciplinary staff training and increased community involvement, can create more compassionate and emotionally safe care settings. Professionals in our study expressed interest in adopting such approaches, recognizing their potential to improve care quality. They emphasized, however, that successful implementation requires strong institutional support, both in material resources and ongoing training, to ensure sustainability and long-term effectiveness.

Adapted communication is a fundamental axis for ensuring child-centered care for children with ASD. Both scientific evidence and participant narratives indicate that effective interaction requires not only sensitivity but also specific skills from healthcare staff. Sabetsarvestani and Geçkil [28] emphasize balancing dyadic and triadic communication (child–nurse–caregiver), using verbal and nonverbal methods, respecting the child’s processing and response times, and avoiding over-demanding interactions or premature interruptions. Tensions also emerged related to administrative aspects affecting care quality. A notable example was the ambiguous management of the *“Doble A”* health card, designed to grant priority and adaptations for individuals with special needs. Although intended to promote preferential treatment, it often generates confusion among professionals, potentially leading to inequalities and frustration among families. This highlights the need to review existing protocols, clarify their application, and provide specific training on differentiated care policies to avoid institutional contradictions and ensure equity in access to services.

This study presents some limitations that should be considered when interpreting its results. First, although the sample size was consistent with theoretical recommendations [17], its relatively small number may have limited the generalizability of the findings and restricted the representation of a broader diversity of experiences. Furthermore, all interviews were conducted by a single interviewer, which ensured methodological consistency but may also have constrained the richness of interactions and introduced potential interpretive biases. To mitigate this risk, strategies to enhance confirmability were applied, including critical review and joint reflection with other researchers, in order to strengthen intersubjectivity and the credibility of the results. Another relevant limitation was the difficulty in recruiting participants, particularly among the parent group, due to their limited availability and willingness to participate. Recruitment of professionals was also challenging, partly because of the limited interest in taking part in the study, which reduced the breadth and diversity of clinical perspectives collected. These recruitment challenges may have introduced selection bias, potentially overrepresenting participants with negative experiences in the pediatric emergency department. For all these reasons, future research should aim to expand the number and variety of participating centers, include larger and more diverse samples, and employ methodological triangulation strategies to strengthen the validity of the findings. Furthermore, it would be pertinent to design and evaluate evidence-based interventions with the aim of analyzing their impact on reducing agitation episodes, improving family satisfaction, and increasing the efficiency of care delivery.

## 5. Conclusions

The findings of this study highlight the urgent need to transform the care of children with ASD in pediatric emergency services, based on the perceptions of both parents and healthcare professionals. Both groups recognize the need for adaptations in pediatric emergency departments to accommodate children with ASD. They agreed on the importance of implementing a sensory-adapted room specifically designed to meet the needs of this population, helping to reduce stress, sensory overload, and episodes of agitation, thereby facilitating more humanized, safe, and effective care. Despite the individual commitment of many professionals, the main barriers identified were a lack of professional training, inadequate hospital environments, and stress experienced by both patients and provider. These factors negatively impact both the quality of care and the experience of families. In this context, implementing concrete measures is considered a priority, including continuous ASD training programs for healthcare staff, improvements to the sensory infrastructure of emergency spaces, and greater flexibility in care protocols. Such actions would support progress toward a more inclusive care model, centered on the actual needs of children with ASD and their families. Furthermore, the results underscore the importance of adopting a comprehensive perspective that considers not only clinical aspects but also the emotional, social, and contextual dimensions shaping the experience of ASD in the hospital environment.

## Figures and Tables

**Table 1:** Sociodemographic characteristics of professionals.

Professional	Gender	Age	Professional Category	Years in Pediatric Emergency Care
Professional 01	Female	39	Pediatric nurse	3
Professional 02	Female	55	Auxiliary nursing care technician	1
Professional 03	Female	60	Pediatric nurse	34
Professional 04	Female	29	Nursing resident-EIR	1
Professional 05	Male	24	Nursing resident-EIR	1
Professional 06	Male	37	Pediatricians	9
Professional 07	Female	31	Auxiliary nursing care technician	4
Professional 08	Female	26	Medical residents pediatricians-MIR	1
Professional 09	Male	26	Medical residents pediatricians-MIR	1
Professional 10	Female	39	Pediatricians	14

**Table 2:** Sociodemographic characteristics of parents.

Parents	Relationship	Age	Employment Status	Number of Children	Age of the Child with ASD *
Parent 01	Mother	47	Employed	2	Son: 16 Daughter: 9
Parent 02	Mother	33	Not employed	2	6
Parent 03	Father	39	Employed	1	5
Parent 04	Mother	35	Not employed	1	8
Parent 05	Mother	49	Employed	4	13
Parent 06	Mother	37	Employed	2	9
Parent 07	Mother	37	Not employed	1	10
Parent 08	Mother	27	Employed	2	5

* ASD: Autism Spectrum Disorder.

**Table 3:** Themes, subthemes, and meaning units.

Participants Group	Themes	Subthemes	Meaning Units
Professionals	Professional Training	Approach Strategies	Intervention techniques, Use of pictograms, Virtual reality, Anesthesia, Clarity, Oral medication, Unnecessary examinations, Coping with trauma, Family collaboration, Nonverbal communication, Effective communication, Unmet needs, Health psychology, Toys, Inclusion of familiar objects, Observation, Touch, Importance of patient care, Healthcare professionals
		Training Received	Self-reflection, Self-taught, Self-assessment, Inadequate training, Collaboration, Knowledge, Prior knowledge, Consideration of other factors, Conviction, Professional development, Lack of knowledge, Discontent, Experience, Lack of training, Insufficient training, Uncertainty, Insecurity, No knowledge, Optimism, Recognition of limitations
		Perceived Needs	Adaptability, Individualized care, Child care, Patient care, Parental consideration, Efficiency of care, Quality of care, Lack of training, Infrastructure, Needs, Time, Material resources, Satisfaction, Conformity, Patient care, Waiting room, Communication difficulties
	Hospital Environment	Resource Allocation	Special care, Shortage, Comfort, Efficiency, Sensory stimulation, Patient needs, Material needs, Pictograms, Space
		Infrastructure	Pleasant environment, Controlled environment, Relaxing environment, Calm environment, Adequate environment, Inadequate environment, Not adapted, Waiting room, Triage, Adapted room
		Perceived Needs During the Emergency Visit	Safe environment, Sensory-friendly environment, Well-being, Care and protection, Healthcare professionals, Audiovisual stimulation, Creative interests, Sensory toys, Light, Sensory needs, Noise, Adapted room, Safety, Personalized treatment
	Emotional Aspects	Emotional Experience Related to Patient Care	Emotional conflict, Aggressiveness, Challenges, Empathy, Stress, Negative experiences, Frustration, Emotional impact, Discomfort, Patience, Patient satisfaction, Emotional sensitivity
		Professionals’ Personal Perceptions	Intervention strategies, Lack of resources, Lack of training, Interaction with children, Infrastructure, Professionalism, Stress, Emergency department, Waiting room
Parents	Professional Training	Perceptions of Staff Training	Anticipation, Family communication, Knowledge, Lack of knowledge, Misinformation, Diversity, Lack of adaptation, Lack of clarity, Lack of communication, Lack of understanding, Lack of training, Lack of updating, Staff training, Language skills, Ignorance, Training, No training
		Demonstrated Emotional Competencies	Adaptation, Awareness, Social awareness, Empathy, Lack of empathy, Lack of humanity, Lack of sensitivity, Misunderstanding by others, Patience, Healthcare professionals, Respect
		Socioemotional Relationships During Care	Conflict, Family conflicts, Challenges, Work-related difficulties, Social avoidance, Lack of adaptation to the child’s needs, Family intervention, Socioemotional judgment, Doctor–patient relationship, Family relationships, Interpersonal relationships, Resistance to rules, Family responsibilities, Sleep disorder, Language disorder
	Hospital Environment	Infrastructure	Accessibility, Child-friendly environment, External environment, Inadequate environment, Restrictive environment, Noisy environment, Environment, Available space, Long waiting time, Impact of the environment, Impact of the environment on patient experience, Importance of a friendly environment in the emergency department, Physical limitation, Needs for a child-friendly environment, Room, Waiting room, Health system, Waiting time, Triage
		Perceived Needs During the Emergency Visit	Sensory stimulation, Auditory stimulus, Visual aid, Tactile stimulus, Visual stimulus, Communication with pictograms, Nonverbal communication, Visual communication, Inadequacy of protocol, Materials, Need, Need for emotional support, Need for training, Need for control, Need for apologies, Need for distraction, Regulated light, Need for more context, Need for recognition, Need for concrete solutions, Specific needs, Medical needs, Adapted needs, Children, Observation, Negative perception of the medical environment, Personalization, Staff preparation, Prioritization of needs, Proposal for improvement, Exclusive protocol, Recognition, Necessary resources, Relaxation, Adapted room, Patient service, Double A card
	Emotional Aspects	Families’ Experiences	Lack of entertainment, Discontent, Fun, Waiting, Parental stress, Negative experience, Positive experience, Lived experience, Functionality, Injustice, Mistreatment, Complaint, Emotional overload
		Emotions During Care	Distress, Anxiety, Family anxiety, Service deficiencies, Poor customer care, Distrust, Desire, Desperation, Difficulty, Disagreement, Hope, Stress, Frustration, Impatience, Helplessness, Uncertainty, Discomfort, Misunderstanding by others, Dissatisfaction, Physical discomfort, Fear, Fear of doctors, Annoyance, Nervousness, Worry, Satisfaction, Feeling of helplessness
	Autism Spectrum Disorder	Diagnostic Characteristics	Attachment, Nonverbal communication, Care, Child care, Atypical development, Language development, Early diagnosis, Communication difficulties, Associated illness, Strategies, Inability to concentrate, Child’s interests, Different needs, Diversified interests, Different needs, Lack of cooperation, Concern for the child’s well-being, Intolerance to noise, Sensory sensitivity, Suspected diagnosis, ASD
		Children’s Needs	Adaptability to patient needs, Individualized care, Family support, Communication with parents, Distraction, Strategies, Lack of visual support, Need for external support, Need for family support, Adapted needs, Children’s needs, Environmental needs, Physical needs, Inadequate needs, Material needs, Parental involvement, Asking what is needed, Occupational therapy
		Sensory Regulation	Self-regulation, Visual aid, Sensory aids, Emotion control, Sensory dysregulation, Distractions, Entertainment, Muscle hyperlaxity, Hypotonia, Mobile phone, Pressure, Reactions to stimuli, Regulation, Emotional regulation, Relaxation techniques, Technology

**Table 4:** Co-occurrence analysis of professionals’ interviews.

Subthemes	A	B	C	D	E	F	G	H
Approach Strategies (A)	0	1	7	2	0	1	7	3
Training Received (B)		0	5	4	0	1	3	1
Perceived Needs (C)			0	1	4	2	5	5
Resource Allocation (D)				0	4	5	3	1
Infrastructure (E)					0	6	2	3
Perceived Needs During the Emergency Visit (F)						0	1	2
Emotional Experience Related to Patient Care (G)							0	1
Professionals’ Personal Perceptions (H)								0

**Table 5:** Co-occurrence analysis of parents’ interviews.

Subthemes	I	J	K	L	M	N	O	P	Q	R
Perceptions of Staff Training (I)	0	11	3	4	11	3	7	8	4	1
Demonstrated Emotional Competencies (J)		0	3	2	11	7	12	6	2	1
Socioemotional Relationships During Care (K)			0	3	2	5	7	4	2	1
Infrastructure (L)				0	11	11	13	7	7	4
Perceived Needs During the Emergency Visit (M)					0	12	17	13	10	7
Families’ Experiences (N)						0	22	8	5	6
Emotions During Care (O)							0	13	5	7
Diagnostic Characteristics (P)								0	7	8
Children’s Specific Needs (Q)									0	5
Sensory Regulation (R)										0

## Data Availability

Data are available upon reasonable request to the corresponding author.

## Supplementary Materials

The following supporting information can be downloaded at: https://www.mdpi.com/article/10.3390/children12091275/s1, Table S1: Semi-structured interview.

## Abbreviations

The following abbreviations are used in this manuscript:

ASD	Autism Spectrum Disorder
COREQ	Consolidated Criteria for Reporting Qualitative Research
DSM-5	Diagnostic and Statistical Manual of Mental Disorders
EIR	nursing residents
HUMIC	Hospital Universitario Materno Infantil de Canarias
MIR	medical residents
MU	Meaning Units
TCAE	auxiliary nursing care technicians
