# Supplementary material for: Adapting Pediatric Emergency Services for Children with Autism Spectrum Disorder: A Phenomenological Approach
Source: Children (Basel). 2025 Sep 22;12(9):1275. doi: 10.3390/children12091275 (PMC12468288; doi:10.3390/children12091275)
Supplement: Supplementary file 1 [file children-12-01275-s001.zip › children-3874533-supplementary.pdf]

Supplementary Table S1: Semi-structured interview.

**Professionals**	1. Have you received specific training on ASD?<br>2. Do you consider that you have sufficient knowledge to adequately care for children with ASD and their families in the emergency department setting?<br>3. Could you describe your experiences attending to children with ASD in the emergency department? Have you faced any particular problems or challenges?<br>4. From your experience, what do you think are the main needs of children with ASD in the emergency context?<br>5. What strategies or resources have you used to interact effectively with children with ASD during care?<br>6. Do you think the hospital has adequate resources to care for this population? If not, what do you think is missing or should be incorporated?<br>7. Are you familiar with the concept of sensory-adapted rooms?<br>8. In your opinion, what materials should a sensory room for children with ASD include?<br>9. Do you believe that the existence of such a room could improve the quality of care offered to these patients? Why?
**Parents**	1. Could you briefly describe your child's clinical situation? (Age, time of ASD diagnosis, language level, presence of comorbidities).<br>2. What was the reason for your recent visit to the pediatric emergency department?<br>3. How would you describe your experience during that visit to the emergency department?<br>4. Were there any initial difficulties related to your child's condition?<br>5. How did your child react to the sensory environment of the department (noise, lights, smells, presence of strangers, etc.)? Was there any part of the environment that caused significant sensory dysregulation?<br>6. Did the healthcare staff demonstrate knowledge about ASD and adopt specific measures to adapt to your child's sensory needs? If yes: What concrete measures were taken? If not: What do you think should have been done?<br>7. What strategies or techniques do you usually use to help your child regulate their sensory sensitivity in everyday situations?<br>8. Were you able to apply any of these strategies during your stay in the emergency department? Did you encounter any obstacles in doing so?<br>9. Are you familiar with sensory-adapted rooms?<br>10. From your perspective, what materials or features should such a room include in order to adequately meet your child's needs?<br>11. Do you think the existence of a sensory-adapted room could have improved your child's experience in the emergency department? Why?
